# The effect of a specialised intervention program for borderline personality disorder on the need for acute hospitalisations: A retrospective observational study

**DOI:** 10.1192/j.eurpsy.2025.1913

**Published:** 2025-08-26

**Authors:** I. da Fonseca Pinto, A. L. Cardoso, A. da Silva Moreira, C. Sousa Reis, H. Ginja, I. Mangas Palma, J. Tavares Coelho, E. Osório, R. Barranha

**Affiliations:** 1 Department of Psychiatry and Mental Health, University Hospital Center of São João; 2 Department of Clinical Neurosciences and Mental Health, Faculty of Medicine of Porto University, Porto, Portugal

## Abstract

**Introduction:**

Borderline personality disorder (BPD) often results in recurrent acute hospitalisations, significantly burdening healthcare systems. Specialised intervention programs aim to stabilise patients and reduce hospital admissions. This study investigates the impact of a structured BPD Intervention Program at the Local Health Unit of São João on reducing the need for acute hospitalisations.

**Objectives:**

The objective of this study is to assess the effect of integration into the BPD Intervention Program on the frequency of acute hospitalizations. Furthermore, we aim to investigate the relationship between clinical and sociodemographic factors, as well as the need for inpatient care.

**Methods:**

This is a retrospective longitudinal study with quantitative statistical analysis of data from 293 patients referred for suspected BPD at the São João Local Health Unit. The analysis included 163 patients who had their BPD diagnosis confirmed. We collected data from hospital records, focusing on the number of hospitalisations before and after program admission. We also analysed variables such as the program’s follow-up duration.

**Results:**

The mean age at the time of the first evaluation was 24.6 years, with symptoms typically starting at a mean age of 14.2 years. The majority of participants (94%) were female. The program’s integration led to a significant reduction in hospital admissions. Prior to the program, patients had an average of 1.01 hospitalisations per patient (median = 0). After integration, the average decreased to 0.66 hospitalisations per patient (median = 0). We are conducting further analyses to explore associations between additional factors and hospitalisation outcomes.

**Conclusions:**

Integration into the BPD Intervention Program is associated with a decrease in acute hospitalizations. This clearly supports the idea that specific interventions can help stabilise BPD patients. The findings also highlight how personalised care can reduce strain on healthcare costs. Future research should evaluate the long-term benefits of the program, including its impact on the duration of hospital stays and the lasting effects of reduced hospitalisation rates after program completion.

**Disclosure of Interest:**

None Declared

